# Low-grade inflammation as a risk factor for cardiovascular events and all-cause mortality in patients with type 2 diabetes

**DOI:** 10.1186/s12933-021-01409-0

**Published:** 2021-11-09

**Authors:** Shahnam Sharif, Y. Van der Graaf, M. J. Cramer, L. J. Kapelle, G. J. de Borst, Frank L. J. Visseren, Jan Westerink, the SMART study group, R. van Petersen, B. G. F. Dinther, A. Algra, Y. van der Graaf, D. E. Grobbee, G. E. H. M. Rutten, F. L. J. Visseren, G. J. de Borst, L. J. Kappelle, T. Leiner, H. M. Nathoe

**Affiliations:** 1 Department of Vascular Medicine, University Medical Center Utrecht, P.O. Box 85500 F02.126, Utrecht, 3508 GA The Netherlands; 2 Julius Center for Health Sciences and Primary Care, University Medical Center Utrecht, Utrecht, The Netherlands; 3 Department of Cardiology, University Medical Center Utrecht, Utrecht, The Netherlands; 4 Department of Neurology, University Medical Center Utrecht, Utrecht, The Netherlands; 5 Department of Vascular Surgery, University Medical Center Utrecht, Utrecht, The Netherlands

## Abstract

**Background:**

Type 2 diabetes is a condition associated with a state of low-grade inflammation caused by adipose tissue dysfunction and insulin resistance. High sensitive-CRP (hs-CRP) is a marker for systemic low-grade inflammation and higher plasma levels have been associated with cardiovascular events in various populations. The aim of the current study is to evaluate the relation between hs-CRP and incident cardiovascular events and all-cause mortality in high-risk type 2 diabetes patients.

**Methods:**

Prospective cohort study of 1679 type 2 diabetes patients included in the Second Manifestations of ARTerial disease (SMART). Cox proportional hazard models were used to evaluate the risk of hs-CRP on cardiovascular events (composite of myocardial infarction, stroke and vascular mortality) and all-cause mortality. Hs-CRP was log-transformed for continuous analyses. Findings were adjusted for age, sex, BMI, current smoking and alcohol use, non-HDL-cholesterol and micro-albuminuria.

**Results:**

307 new cardiovascular events and 343 deaths occurred during a median follow-up of 7.8 years (IQR 4.2–11.1). A one unit increase in log(hs-CRP) was related to an increased vascular- and all-cause mortality risk (HR 1.21, 95% CI 1.01–1.46 and HR 1.26, 95% CI 1.10–1.45 respectively). No relation was found between log(hs-CRP) and myocardial infarction or stroke. The relations were similar in patients with and without previous vascular disease.

**Conclusion:**

Low grade inflammation, as measured by hs-CRP, is an independent risk factor for vascular- and all-cause mortality but not for cardiovascular events in high-risk type 2 diabetes patients. Chronic low-grade inflammation may be a treatment target to lower residual cardiovascular risk in type 2 diabetes patients.

**Supplementary Information:**

The online version contains supplementary material available at 10.1186/s12933-021-01409-0.

## Background

Cardiovascular disease is the leading cause for hospital admissions and mortality in patients with type 2 diabetes [1]. Patients with type 2 diabetes are at higher risk of cardiovascular morbidity and mortality compared to patients without type 2 diabetes [2–4]. Most of the observed increased cardiovascular risk is attributed to clustering of known cardiovascular risk factors (e.g. hypertension, dyslipidemia and hyperglycemia), but even when treated according to international cardiovascular prevention guidelines, a substantial residual cardiovascular risk still remains in which low grade inflammation may play an important role [5].

Insulin resistance is a hallmark in patients with type 2 diabetes leading not just to hyperglycemia but also to low-grade inflammation. When excess adipose tissue becomes dysfunctional, it produces pro-inflammatory cytokines like tumor necrosis factor alpha (TNF-α) and interleukin-6 (IL-6) while the release of anti-inflammatory adipokines such as adiponectin is reduced [6]. The inflammatory adipocytokines TNF-α and IL-6 are related to elevated levels of circulating C-reactive protein (CRP) [7–9]. These changes in the release of adipocytokines, from especially visceral adipose tissue, induces a state of systemic insulin resistance and low-grade inflammation [6]. Indeed, in patients without diabetes, elevated levels of CRP are related to future insulin resistance and development of type 2 diabetes [10].

Proposed mechanisms through which low-grade inflammation might influence atherogenesis include accelerated foam cell formation by monocyte recruitment and native low-density lipoprotein cholesterol (LDL-c) uptake in the arterial wall [11, 12] Elevated CRP levels are also associated with blunted systemic endothelial vasodilator function and atherothrombosis is promoted via inducement of plasminogen activator inhibitor-1 expression and activity in human endothelial cells [13, 14].

In various populations low-grade inflammation has indeed been related to a higher risk of cardiovascular events and cardiovascular mortality [15–17]. In acute myocardial infarction patients with and without DM, hs-CRP predicts in-hospital outcome and two-year mortality [18]. It has also been shown that lowering low-grade inflammation in patients with coronary artery disease with or without type 2 diabetes reduces the residual risk for cardiovascular events [19]. In patients with type 2 diabetes a relation between higher plasma levels of high-sensitivity C-reactive protein (hs-CRP) and non-fatal and fatal cardiovascular events have been shown [20, 21]. In contrast however, in a case-cohort study nested within a type 2 diabetes trial (ADVANCE trial), there was no relation between hs-CRP levels and the risk of macro-and microvascular complications nor with the risk of death [22].

The aim of the present study is to evaluate the relation between hs-CRP plasma levels and cardiovascular events and all-cause mortality in high-risk type 2 diabetes patients with and without clinical manifest vascular disease.

## Methods

### Study population

The Second Manifestations of ARTerial disease (SMART) study is an ongoing prospective cohort at the University Medical Center Utrecht, The Netherlands. A rationale and detailed description of the SMART study has been previously published [23]. Patients between ages 18–79, who were newly referred to the University Medical Center Utrecht with clinical manifest vascular disease or with important risk factors for atherosclerotic disease (e.g. diabetes, hyperlipidemia or hypertension), are asked to participate. At inclusion, information is obtained through questionnaires on medical history, history of vascular disease (coronary artery disease, cerebrovascular disease, peripheral arterial disease, abdominal aortic aneurysm), cardiovascular risk factors (e.g. hypertension, hyperlipidemia, smoking, alcohol consumption, physical activity) and medication use. Blood pressure was measured using a semiautomatic oscillometric device during 25 min in sitting position, three times at both upper arms with the highest mean of the last two measurements on one arm taken as the blood pressure. Height and weight were measured without shoes and in light clothing and are used to calculate body mass index (BMI).

A detailed description of the specific laboratory measurements can also be found in the previously mentioned rationale [23]. To summarize, blood samples were collected in a fasting state to measure hs-CRP, hemoglobin A1c (HbA1c), glucose, kidney function estimated by Chronic Kidney Disease Epidemiology Collaboration (CKD-EPI) equation and serum lipids: total cholesterol, high-density lipoprotein cholesterol (HDL-c), triglycerides (TG). LDL-c was calculated using the Friedewald formula up to plasma triglyceride levels of 9 mmol/l. A urine sample was also collected to measure albuminuria.

The study was approved by the Medical Ethics Committee of the University Medical Center Utrecht and informed consent was obtained from all participants.

The current study uses data from patients enrolled in SMART diagnosed with type 2 diabetes (n = 1910). Before the year 2003, hs-CRP was not measured in a standardized manner and therefore hs-CRP was missing in 305 patients. These values were assumed to be missing at random and therefore single imputation methods were used to reduce missing data. To rule out inflammation due to other causes besides low-grade inflammation, all hs-CRP values above 10 mg/L were excluded, leaving 1679 patients for analysis.

### Follow-up

Biannual questionnaires were used to obtain information on outpatient clinic visits and hospitalization. All available data on reported events was collected. Death was reported either by relatives, the general practitioner, or treating specialist. Three members of the SMART study Endpoint Committee independently evaluated all reported events. Primary outcomes for the current study were a composite of major adverse cardiovascular events (MACE: consisting of myocardial infarction (MI), stroke and vascular mortality), and all-cause mortality.

MI was defined as at least two of the following criteria: (i) Chest pain for at least 20 min, not disappearing after administration of nitrates (ii) ST-elevation > 1 mm in two following leads on ECG or a Left Bundle Branch Block (iii) Cardiac enzyme elevation (Troponin above clinical cut-off value or creatinine kinase (CK) of at least two times the normal value and a myocardial band fraction > 5% of the total CK. Sudden cardiac death was also considered as MI.

A definite stroke was diagnosed in case of relevant clinical features causing an increase in impairment of at least one grade on the modified Rankin scale, accompanied by an infarction of hemorrhage on a repeat CT‐scan. A probable stroke was diagnosed in case of clinical deficits causing an increase in impairment of at least one grade in the modified Rankin scale, without CT-scan documentation.

Vascular mortality was defined as death due to MI, stroke, congestive heart failure, rupture of abdominal aortic aneurysm and vascular death from other causes including heart failure.

All-cause mortality was defined as death due to any cause.

The period between patient inclusion and first cardiovascular event, death, loss to follow-up or the predefined date of March 2015 was defined as the follow-up duration. In total, 124 patients (7.4%) were lost to follow-up due to relocation or discontinuation of the study.

### Data analyses

The baseline characteristics are presented in quartiles of hs-CRP in order to visualize potential differences in baseline characteristics in patients with different levels of hs-CRP.

As missing data and complete case analysis can lead to bias, single imputation methods were used to reduce missing covariate data for non-high-density lipoprotein cholesterol (non-HDL-c) (n = 15; 0.9%), current smoking (n = 16; 1.0%), alcohol use (n = 19; 1.1%), and albuminuria (n = 115; 6,8%).

The relation between hs-CRP and cardiovascular events and all-cause mortality was evaluated by Cox proportional-hazard models. Due to its right skewed distribution, hs-CRP was analyzed after logarithmic transformation and in tertiles. Hazard ratios (HR) and 95% confidence intervals (95%CI) were calculated for hs-CRP as a continuous variable per 1 unit increase in log(hs-CRP) mg/l and as a categorical variable using tertiles of hs-CRP with the first tertile as reference. Three models were used. In model I the relation between hs-CRP and the endpoint of interest was adjusted for age and sex. In model II additional adjustment was performed for body mass index, current smoking, alcohol, non-HDL-cholesterol and micro-albuminuria. Model III is an exploratory model for additional adjustment of lipid-lowering therapy and aspirin use as they might have anti-inflammatory properties and therefore influence hs-CRP values [15, 24–26]. The variables in the first two models are a set of traditional cardiovascular risk factors which are known to be related to inflammation based on literature. The proportional hazards assumption was assessed by plotting and visual inspection of the Schoenfeld residuals and showed no signs of violation. Linear and non-linear relations were explored using restricted cubic splines.

To evaluate whether the relation between hs-CRP and cardiovascular events or all-cause mortality was different for patients with or without manifest vascular disease a multiplicative interaction term was included in the Cox models and analyses were performed after stratification for cardiovascular disease. Effect modification was considered to be present if the p-value of the interaction term was < 0.05.

Analyses were performed using statistical package R 3.2.2 and for all analyses a p-value < 0.05 was considered statistically significant.

## Results

### Baseline characteristics

The baseline characteristics are presented in tertiles of log(hs-CRP) in Table 1. Over the tertiles of log(hs-CRP), the percentage of men and current alcohol use decreased, while current smoking increased and age, systolic, and diastolic blood pressure were similar. Glucose, HbA1c, LDL-cholesterol, triglycerides and non-HDL-cholesterol all increased over the tertiles, while the estimated glomerular filtration rate (eGFR) decreased.

**Table 1:** Baseline characteristics according to tertiles of hs-CRP

Tertiles of hs-CRP N = 1679	Tertile 1 n = 558	Tertile 2 n = 561	Tertile 3 n = 560	p-value
Range hs-CRP, mg/l	0.13–1.35	1.35–3.22	3.22–9.97	
Men, n (%)**	434 (78)	400 (71)	361 (65)	< 0.05
Age (years)*	61 (10)	61 (10)	60 (10)	0.11
Hypertension*	360 (65)	401 (71)	397 (71)	0.07
Smoking current, n (%)**	80 (14)	129 (23)	188 (34)	< 0.05
Pack-years***	9 IQR(0–26)	13 IQR(0–31)	16 IQR(2–33)	< 0.05
Current alcohol use, n (%)**	324 (58)	258 (46)	213 (38)	< 0.05
Duration of diabetes (years)***	4 IQR(1–10)	4 IQR(1–10)	4 IQR(0–9)	0.20
BMI (kg/m^2^)*	28 (4)	29 (5)	30 (5)	0.19
Systolic blood pressure, mmHg*	144 (21)	146 (22)	146 (20)	0.33
Diastolic blood pressure, mmHg*	83 (12)	83 (12)	83 (11)	0.86
Laboratory measurements				
Glucose, mmol/l*	8.4 (2.6)	8.6 (2.9)	8.9 (3.1)	< 0.05
HbA1c, %*	6.8 (1.1)	7.1 (1.3)	7.2 (1.3)	< 0.05
eGFR, ml/min/1.73 m^2*^	80.3 (19.8)	79.5 (23.0)	77.5 (21.6)	0.06
Micro-albuminuria, n (%)**	103 (20)	128 (24)	118 (23)	0.31
Total Cholesterol, mmol/l*	4.5 (1.2)	4.9 (1.4)	5.0 (1.4)	< 0.05
LDL-c, mmol/l*	2.5 (1.0)	2.8 (1.1)	2.9 (1.1)	< 0.05
HDL-c, mmol/l*	1.2 (0.4)	1.1 (0.3)	1.1 (0.3)	< 0.05
non-HDL-c, mmol/l*	3.3 (1.2)	3.4 (1.4)	3.9 (1.4)	< 0.05
Triglycerides, mmol/l***	1.5 IQR(1.0–2.2)	1.7 IQR(1.2–2.5)	1.8 IQR(1.3–2.7)	< 0.05
Type of vascular disease				
Coronary artery disease, n (%)**	270 (48)	269 (48)	226 (40)	< 0.05
Cerebrovascular disease, n (%)**	101 (18)	97 (17)	125 (22)	< 0.05
Peripheral artery disease, n (%)**	60 (11)	74 (13)	89 (16)	< 0.05
Abdominal Aortic Aneurysm, n (%)**	21 (4)	27 (5)	29 (5)	0.32

*BMI* body mass index, *eGFR* estimated Glomerular Filtration Rate by the CKD-EPI equation, *HDL-c* high-density lipoprotein cholesterol, *LDL-c* low-density lipoprotein cholesterol

*Continuous variables are depicted as mean (SD), **count variables as n (%) and ***not normally distributed variables as median IQR

### Relation between hs-CRP and risk of cardiovascular events

A total of 307 new cardiovascular events occurred during a total follow-up of 15.9 years (median 7.8 years, IQR 4.2–11.1). The causes of death are presented in tertiles of log(hs-CRP) in Additional file 1: Table S1. When analyzed categorically using the first tertile as reference, no relation was found for myocardial infarction (HR 1.23, 95% CI 0.85–1.85), stroke (HR 0.67, 95% CI 0.37–1.20) or cardiovascular events (HR 1.24, 95% CI 0.91–1.68) (Table 2). When analyzed per one unit increase in log(hs-CRP), again no relation was found for myocardial infarction (HR 1.14, 95%CI 0.94–1.38), stroke (HR 0.83, 95% CI 0.64–1.08) or cardiovascular events (HR 1.08, 95% CI 0.94–1.25) (Table 3). Subgroup analyses did not support effect modification by presence of manifest vascular disease (all p-values for interaction > 0.05). Additional adjustment for lipid-lowering therapy and aspirin use did not change the results (Tables 2 and 3).

**Table 2:** Relation between hs-CRP and cardiovascular events and mortality in tertiles of hs-CRP

Tertiles of log(hs-CRP)	Tertile 1	Tertile 2	Tertile 3
n = 1679	n = 558	n = 561	n = 560
Range hs-CRP, mgl/L	0.13–1.35	1.35–3.22	3.22–9.97
Myocardial infarction			
n	41	53	62
Model I	1.00 (ref)	1.22 (0.81–1.83)	1.46 (0.98–2.18)
Model II	1.00 (ref)	0.91 (0.59–1.39)	1.07 (0.70–1.63)
Model III	1.00 (ref)	0.87 (0.57–1.32)	1.23 (0.81–1.85)
Stroke			
n	24	33	28
Model I	1.00 (ref)	1.28 (0.76–1.17)	1.12 (0.76–2.17)
Model II	1.00 (ref)	0.96 (0.55–1.68)	0.81 (0.45–1.47)
Model III	1.00 (ref)	0.71 (0.40–1.22)	0.67 (0.37–1.20)
Cardiovascular events			
n	80	102	125
Model I	1.00 (ref)	1.19 (0.88–1.59)	1.52 (1.14–2.02)
Model II	1.00 (ref)	0.93 (0.68–1.27)	1.16 (0.85–1.57)
Model III	1.00 (ref)	0.88 (0.65–1.21)	1.24 (0.91–1.68)
Vascular mortality			
n	38	58	88
Model I	1.00 (ref)	1.47 (0.98–2.22)	2.34 (1.59–3.43)*
Model II	1.00 (ref)	1.26 (0.81–1.95)	1.87 (1.23–2.84)*
Model III	1.00 (ref)	1.37 (0.89–2.10)	1.87 (1.22–2.85)*
All-cause mortality			
n	74	113	156
Model I	1.00 (ref)	1.47 (1.09–1.97)*	2.11 (1.59–2.79)*
Model II	1.00 (ref)	1.26 (0.92–1.72)	1.72 (1.27–2.33)*
Model III	1.00 (ref)	1.34 (0.98–1.84)	1.89 (1.39–2.57)*

hsCRP per tertiles, T1 = ref, hazard ratio (95% confidence interval); *statistically significant p < 0.05

Model I: age + sex

Model II: Model I + BMI, smoking, alcohol, non-HDL-c, micro-albuminuria

Model III: Model II + lipid-lowering therapy, aspirin

**Table 3:** Relation between log(hs-CRP) and cardiovascular events and mortality

	Total population	Without manifest vascular disease	With manifest vascular disease
	n = 1679	n = 481	n = 1124
	HR (95% CI)	HR (95% CI)	HR (95% CI)
Myocardial infarction	n = 156	n = 25	n = 131
Model I	1.16 (0.97–1.39)	1.27 (0.77–2.09)	1.21 (1.00–1.48)
Model II	1.03 (0.85–1.25)	1.14 (0.67–1.93)	1.07 (0.86–1.32)
Model III	1.14 (0.94–1.38)	1.15 (0.68–1.96)	1.07 (0.86–1.33)
Stroke	n = 85	n = 16	n = 61
Model I	1.02 (0.81–1.30)	0.93 (0.53–1.62)	1.03 (0.93–1.15)
Model II	0.89 (0.68–1.15)	0.70 (0.38–1.29)	0.89 (0.65–1.22)
Model III	0.83 (0.64–1.08)	0.69 (0.37–1.28)	0.91 (0.66–1.25)
Cardiovascular events	n = 307	n = 50	n = 246
Model I	1.16 (1.02–1.33)*	1.11 (0.79–1.56)	1.18 (1.03–1.37)*
Model II	1.03 (0.90–1.19)	0.97 (0.67–1.39)	1.06 (0.91–1.25)
Model III	1.08 (0.94–1.25)	0.99 (0.68–1.44)	1.07 (0.91–1.25)
Vascular mortality	n = 184	n = 29	n = 154
Model I	1.36 (1.14–1.62)*	1.20 (0.77–1.86)	1.38 (1.14–1.66)*
Model II	1.20 (1.00–1.45)*	1.18 (0.73–1.93)	1.18 (0.95–1.45)
Model III	1.21 (1.01–1.46)*	1.24 (0.75–2.06)	1.18 (0.95–1.46)
All-cause mortality	n = 343	n = 64	n = 270
Model I	1.10 (1.06–1.13)*	1.26 (0.94–1.69)	1.11 (1.06–1.17)*
Model II	1.20 (1.04–1.37)*	1.06 (0.95–1.18)	1.20 (1.04–1.37)*
Model III	1.26 (1.10–1.45)*	1.17 (0.83–1.63)	1.21 (1.03–1.41)*

hs-CRP continuous, HR per 1 mg/l increase, hazard ratio (95% confidence interval)

*statistically significant p < 0.05

Model I: age + sex

Model II: Model I + BMI, smoking, alcohol, non-HDL-c, micro-albuminuria

Model III: Model II + lipid-lowering therapy, aspirin

### Relation between hs-CRP and mortality

A total of 343 deaths occurred during a total follow-up of 15.9 years (median 7.8 years, IQR 4.2–11.1). When analyzed categorically, patients in the third tertile had an 89% higher risk of mortality than patients in the first tertile (HR 1.89, 95% CI 1.39–2.57) (Table 2). Patients in the third tertile were also at increased vascular mortality risk as compared with patients in the first tertile (HR 1.87, 95% CI 1.22–2.85) (Table 2). When analyzed continuously, a one unit increase in log(hs-CRP) was related to an increased mortality risk (HR 1.26, 95% CI 1.10–1.45) (Table 3). A one unit increase in log(hs-CRP) was also related to an increased vascular mortality risk (HR 1.21, 95%CI 1.01–1.46) (Table 3). Subgroup analyses did not support effect modification by presence of manifest vascular disease (all p-values for interaction > 0.05). Furthermore, additional adjustment for lipid-lowering therapy and aspirin use did not change the results (Tables 2 and 3).

## Discussion

In patients with type 2 diabetes with and without manifest vascular disease, every 1 mg/l increase in plasma hs-CRP is related to a 21% increased risk of vascular mortality and 26% increased risk of all-cause mortality. Plasma hs-CRP levels were not related to cardiovascular events in patients with type 2 diabetes mellitus.

### Relation to previous studies

The findings of the current study are partially in line with previous studies that assessed the relation between hs-CRP and cardiovascular events. In an apparent healthy population, hs-CRP had already been found to be related with increased risk of myocardial infarction and stroke [15, 16]. Healthy men in the highest quartile of hs-CRP levels had a 3 times higher risk of myocardial infarction and a 2 times higher risk of stroke as compared to healthy men in the lowest quartile [15]. Women in the highest quartile had a five-fold increase in risk of cardiovascular events [16]. In the present study we did not find any relation between hs-CRP levels and cardiovascular events such as myocardial infarction and stroke. An explanation for this discrepancy might lie within the difference in study population which consisted of type 2 diabetes patients already at high-risk of cardiovascular morbidity and mortality in contrast to a healthy population, as the relation between hs-CRP and cardiovascular morbidity might differ for a patient with or without type 2 diabetes. To illustrate, only patients at cardiovascular risk receive lipid-lowering therapy which may have anti-inflammatory effects and therefore affect CRP levels while also reducing cardiovascular risk by lowering LDL-cholesterol [24–26].

In a large Chinese prospective study, patients with type 2 diabetes without vascular disease were identified through health examinations among employees of Kai Lan Group. Participants in the highest quartile of hs-CRP were found to be at increased risk of a first myocardial infarction or cerebral infarction as compared to patients in the lowest quartile [27]. Again an explanation for the discrepancy in results might lie within the study population as the current study consisted of high-risk type 2 diabetes patients referred to a tertiary center in contrast to asymptomatic type 2 diabetes patients identified during health examinations. In another study among patients with type 2 diabetes, higher plasma levels of hs-CRP were independently associated with non-fatal cardiovascular events [20]. However, in contrast to the current study, the definition of non-fatal cardiovascular events also included transient ischemic attacks and stable or unstable angina. These “soft” clinical endpoints are subject to bias as they require subjective assessment by investigators and patients and including these in a composite endpoint definition may influence the results.

In two previous large population-based cohort studies, higher levels of hs-CRP were also related to higher risk of cardiovascular mortality, in line with our current findings [28, 29]. Both of the studies found no difference in results for patients with or without diabetes. However, in contrast to the current study, the study populations consisted of a sample of the general population with and without diabetes. No distinction was made between type 1 and type 2 diabetes and no information was provided on the presence of manifest vascular disease. In contrast, the current study is comprised of solely high risk type 2 diabetes patients who were referred to a tertiary center. It is especially in this high-risk group of patients that Inflammation is considered to be an additional treatment target to reduce residual cardiovascular risk. The current study adds to the existing evidence demonstrating that in this particular high-risk group of type 2 diabetes patients, inflammation is a risk factor for vascular mortality and that the results also hold for high-risk type 2 diabetes patients with and without manifest vascular disease.

Our findings are in contrast to the results from a post-hoc analysis in the Action in Diabetes and Vascular Disease: Preterax and Diamicron Modified Release Controlled Evaluation (ADVANCE) population. In this nested case-cohort study, hs-CRP was related to a composite of fatal and non-fatal macrovascular events but after adjustment for interleukin-6, the relation was abolished. Instead interleukin-6 was found to be a significant independent predictor of macrovascular events and mortality in this study including patients with type 2 diabetes [22]. It is however debatable whether interleukin-6 should be adjusted for in a study with hs-CRP as the determinant, as it might be in the causal pathway leading from adipose tissue dysfunction to low-grade inflammation [30].

Increased hs-CRP plasma levels have also been related to increased mortality risk due to coronary heart disease in a cohort of type 2 diabetes patients [21]. Patients with hs-CRP plasma levels above 3 mg/l had an increased coronary heart disease mortality risk of 72% compared to patients with hs-CRP plasma levels below 3 mg/l. This is in accordance with the findings of the current study as patients with hs-CRP plasma levels above 3,2 mg/l corresponding to the third tertile were found to have an increased vascular mortality risk of 87% compared to patients with hs-CRP plasma levels of below 1.3 mg/l (corresponding to the first tertile). Our results are similar to those found in the Casale Monferrato Study in which in type 2 diabetes patients with hs-CRP levels above 3 mg/l, an increased short term mortality risk was found when compared to patients with hs-CRP levels below 3 mg/l [31].

Additionally, as mentioned before it has been stated that lipid-lowering therapy may have anti-inflammatory effects and may therefore affect CRP levels and reduce cardiovascular risk [24–26]. At least 50% of patients in the current study used lipid-lowering therapy which may have influenced the results. However as can be seen in the exploratory model of the analyses where additional adjustment was made for lipid-lowering therapy and aspirin use, the magnitude and direction of the estimates did not change and therefore the effect of lipid-lowering therapy can be dismissed.

### Strengths & limitations

Strengths of this cohort study include a data collection in a standardized manner, a substantial number of cardiovascular end points and an endpoint committee which independently evaluated the endpoints. Study limitations need to be considered, including the fact that only baseline data was available, knowing that hs-CRP and risk factor levels may have changed over the course of follow-up. Multiple measurements of hs-CRP and major covariates are preferred to be incorporated as time-varying variables in the Cox proportional hazards regression models. Also, after stratification, the group of patients without manifest vascular disease resulted in a small group of patients with a low number of events.

Another limitation we have to consider is the effect of smoking. As can been seen in the baseline table, the amount of current smokers increased with each tertile of hs-CRP levels. Smoking is therefore a possible confounder as there is a clear relationship with cardiovascular endpoints and a presumable relationship with hs-CRP and by extent inflammation. Smoking was considered a confounder at forehand and was therefore included in the statistical models in order to account for smoking. As such, a possible confounding effect is considered minimal.

### Implications

The results of this study add to the evidence to show that low-grade inflammation plays an important role in the pathogenesis of cardiovascular disease in patients with type 2 diabetes. Whether low-grade inflammation in general can be used as a therapeutical target, has been recently evaluated in the Canakinumab Anti-Inflammatory Thrombosis Outcome Study (CANTOS) trial [19]. The results showed that anti-inflammatory therapy targeting the interleukin-1β innate immunity pathway led to a lower rate of cardiovascular events in patients with a previous myocardial infarction. Although the CANTOS study population was not comprised of specifically type 2 diabetes patients, approximately 40% of the patients did have type 2 diabetes. The CANTOS study was also included in a recently published meta-analysis evaluating anti-inflammatory therapy on major cardiovascular events in patients with diabetes [32]. The meta-analysis included a total of five studies: four studies that used colchicine and the cantos study. The conclusion was that the use of anti-inflammatory therapy in patients with type 2 diabetes and atherosclerotic cardiovascular disease was associated with reduced risk of major cardiovascular events. This would suggest that the inflammatory pathway could be considered a potential therapeutic target in patients with type 2 diabetes.

## Conclusion

In conclusion, low-grade inflammation, as measured by hs-CRP, is an independent risk factor for vascular- and all-cause mortality, in high-risk type 2 diabetes patients with and without manifest vascular disease.

## Supplementary Information

**Additional file 1: Table S1**. Causes of mortality according to tertiles of hs-CRP.

## Data Availability

The datasets generated and/or analyzed during the current study are not publicly available due to individual patient data and privacy, but may be available from the corresponding author on reasonable request.

## Abbreviations

95% CI: 95% Confidence intervals
ADVANCE: Action in Diabetes and Vascular Disease: Preterax and Diamicron Modified Release Controlled Evaluation trial
BMI: Body mass index
CANTOS: Canakinumab Anti-Inflammatory Thrombosis Outcome Study trial
CKD-EPI: Chronic Kidney Disease Epidemiology Collaboration
CK: Creatinine kinase
CRP: C-reactive protein
eGFR: Estimated glomerular filtration rate
HbA1c: Hemoglobin A1c
HDL-c: High-density lipoprotein cholesterol
HR: Hazard ratios
hs-CRP: High-sensitivity C-reactive protein
IL-6: Interleukin-6
IQR: Inter quartile range
LDL-c: Low-density lipoprotein cholesterol
MACE: Major adverse cardiovascular events
MI: Myocardial infarction
non-HDL-c: Non-high-density lipoprotein cholesterol
SMART: Second Manifestations of ARTerial disease study
TG: Triglycerides
TNF-α: Tumor necrosis factor alpha
