# Supplementary material for: Low-grade inflammation as a risk factor for cardiovascular events and all-cause mortality in patients with type 2 diabetes
Source: Cardiovasc Diabetol. 2021 Nov 9;20:220. doi: 10.1186/s12933-021-01409-0 (PMC8579639; doi:10.1186/s12933-021-01409-0)
Supplement: Supplementary file 1 — Additional file 1: Table S1. Causes of mortality according to tertiles of hs-CRP. [file 12933_2021_1409_MOESM1_ESM.docx]

# Additional file 1: Table S1. Causes of mortality according to tertiles of hs-CRP

| Tertiles of log(hs-CRP)  n=1679  Range hs-CRP, mgl/L | Tertile 1  n=558  0.13 – 1.35 | | Tertile 2  n=561  1.35 - 3.22 | | Tertile 3  n=560  3.22 - 9.97 | |
| --- | --- | --- | --- | --- | --- | --- |
|  |  |  |  |  |  |  |
| Causes of mortality |  |  |  |  |  |  |
| Total death | 78 | **(14%)** | 110 | **(20%)** | 155 | **(28%)** |
|  |  |  |  |  |  |  |
| Vascular | 39 | **(7%)** | 61 | **(11%)** | 87 | **(16%)** |
| Malignancy | 26 | **(5%)** | 24 | **(4%)** | 39 | **(7%)** |
| Infection | 4 | **(1%)** | 7 | **(1%)** | 10 | **(2%)** |
| Other non-vascular | 6 | **(1%)** | 8 | **(1%)** | 4 | **(1%)** |
| Unnatural causes | 0 | **(0%)** | 2 | **(0%)** | 1 | **(0%)** |
| No classification | 3 | **(1%)** | 8 | **(1%)** | 14 | **(3%)** |
|  |  |  |  |  |  |  |
